# Effectiveness and predictors of conversion in mini-laparotomy cholecystectomy in developing country: a cohort retrospective study

**DOI:** 10.1186/s12893-022-01792-9

**Published:** 2022-09-19

**Authors:** Adeodatus Yuda Handaya, Joshua Andrew, Ahmad Shafa Hanif, Kevin Radinal Tjendra, Azriel Farrel Kresna Aditya

**Affiliations:** 1 Digestive Surgery Division, Department of Surgery, Faculty of Medicine, Public Health, and Nursing, Universitas Gadjah Mada/Dr, Sardjito Hospital, Jl. Kesehatan No. 1, Yogyakarta, 55281 Indonesia; 2 Faculty of Medicine, Public Health, and Nursing, Universitas Gadjah Mada, Yogyakarta, 55281 Indonesia

**Keywords:** Cholecystitis, Cholelithiasis, Conversion surgery, Mini-laparotomy cholecystectomy, Predictors of conversion

## Abstract

**Background:**

Mini laparotomy cholecystectomy (MLC) is an alternative surgical procedure in conditions where laparoscopic cholecystectomy (LC) is not feasible. MLC is a simpler and easier technique compared to LC. MLC involves smaller skin incision, low morbidity rate, and early return to oral diet. MLC has the potential to be the preferred surgical technique in developing countries due to its low cost and availability.

**Method:**

A cohort retrospective study was performed on 44 patients who underwent mini laparotomy cholecystectomy due to ineligibility for LC. Patients were documented for successful mini laparotomy or conversion to laparotomy cholecystectomy. There are pre-operative aspects recorded and analyzed to formulate predictor factors for conversion surgery, as well as intra-operative and post-operative aspects. Patients also filled evaluation questionnaire based on Likert Scale about their satisfaction towards result of MLC.

**Result:**

MLC is performed in 31 (70.5%) patients while 13 (29.5%) patients underwent conversion to open cholecystectomy. There were no complications nor mortalities observed during and after the surgery. Greater BMI, higher leucocyte count, higher bilirubin level, increasing severity of adhesion, and chronic cholecystitis were found to be statistically significant (p < 0.05) in the conversion surgery group. MLC also resulted in shorter post-operative hospitalization compared to conversion surgery. Patients showed great satisfaction towards the cosmetic aspect and recovery period after MLC procedure.

**Conclusion:**

MLC is an effective surgery procedure for cholelithiasis and can be safely performed in patients with complication such as cholecystitis and gallbladder adhesion although these conditions increase the risk of conversion surgery.

## Background

Cholelithiasis is a very common disease in hepatobiliary surgery. Currently, its prevalence is constantly rising with the improvement of living standard of people. The incidence of cholelithiasis is as high as 10–15% of the adult population [1, 2]. Cholelithiasis can manifest no symptoms while some can cause unbearable pain. Patients with symptomatic gallstones should be offered surgical procedure such as open cholecystectomy and laparoscopic cholecystectomy unless the patient is medically unfit for surgery [2]. Laparoscopic cholecystectomy (LC) has become the gold standard in the treatment of symptomatic gallstones in the last two decades. However, in developing countries, LC is not widely available as it is an expensive operation due to the required high technological equipment [3].

**Fig. 1:**
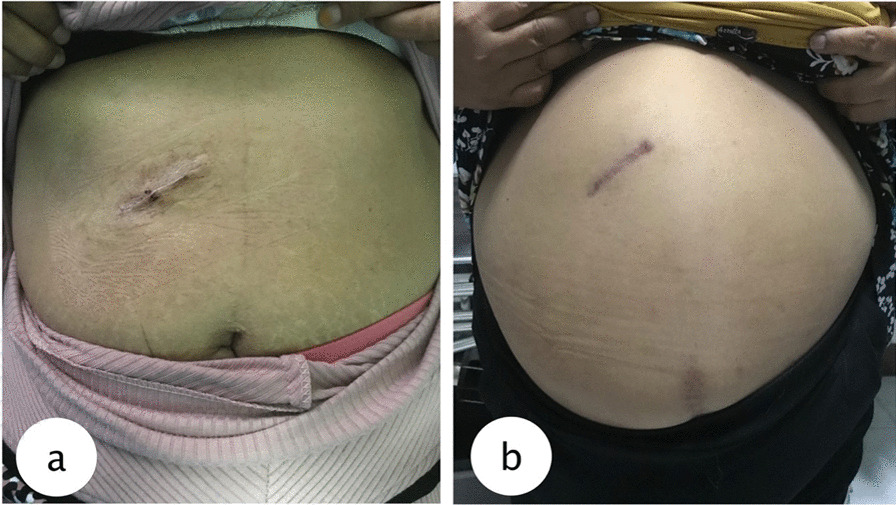
Post operative wound after mini-laparotomy cholecystectomy. **a** On a normal weight patient. **b** on an obese patient

**Fig. 2:**
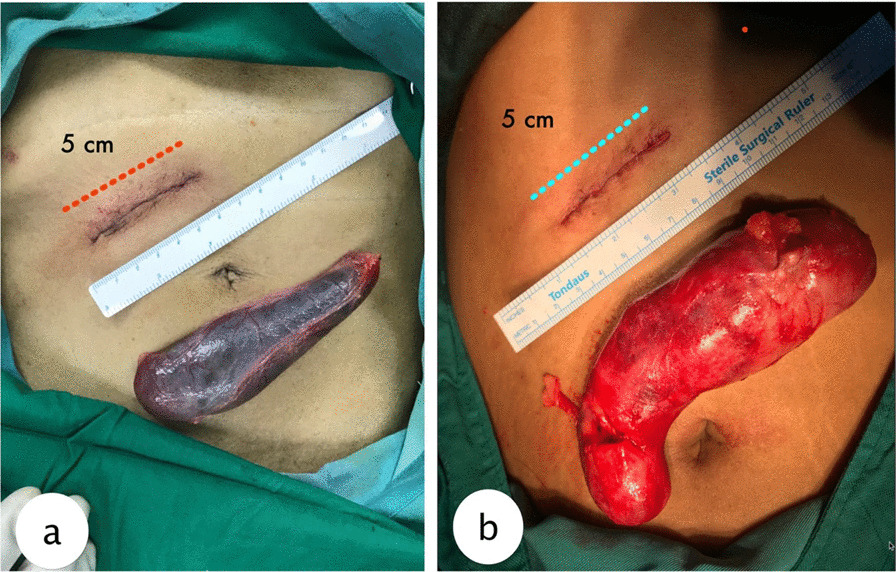
Side by side comparison of the size of dissected gallbladder and the mini-laparotomy incision. **a** gallbladder without inflammation. **b** gallbladder with inflammation

Mini-laparotomy cholecystectomy (MLC), which involves much smaller skin incision, is currently increasing in popularity as an alternative surgical method for definite treatment of cholelithiasis, as it provides low morbidity rate, an early return to oral diet, requiring only few doses of postoperative analgesic unlike the conventional laparotomy, simple, and an easy to learn technique [3, 4]. The principle of the technique is to minimizes physical trauma to the operated patient. According to Shulutko et al., MLC technique involves a 3–5 cm longitudinal incision around 4 cm lateral to the midline at the subcostal margin (Figs. 1a, b and 2a, b). The surgery technique does not require neither the surgeon’s hand nor even his finger from entering the abdominal cavity, thus further minimising the risk of exposure to contaminants [4]. Unlike laparoscopic surgeries, mini-laparotomy do not require extra expenses for the equipment and extra special trainings to operate the laparoscopic equipment. Therefore, mini-laparotomy cholecystectomy can be deemed less costly than laparoscopic cholecystectomy. Many studies reported similar satisfaction and similar length of hospital stay for the two operations which makes both operations equally feasible as a treatment of choice in cholelithiasis patients [4].

Indonesia is a developing country where the government had just recently imposed a national health insurance and thus creating a price ceiling for each medical treatment. This situation put an inclination for performing MLC in cases of cholelithiasis with or without cholecystitis. This study was conducted to evaluate the effectiveness and predictors of conversion in mini-laparotomy cholecystectomy.

## Methods

This study was a cohort retrospective study. We observed medical record of 44 patients who underwent mini laparotomy cholecystectomy on July 20th, 2018, to July 9th, 2019 by a surgeon with 500 mini laparotomy cholecystectomy and open cholecystectomy experiences combined. The patients were documented for successful mini laparotomy or converted to laparotomy cholecystectomy. Both groups were compared in pre-operative aspects: (1) sex, (2) age, (3) Body Mass Index (BMI), (4) cholecystitis clinical manifestation (abdominal pain, nausea—vomiting, fever, Murphy’s sign, icterus), (5) hemoglobin level, (6) leucocytes count, (7) Neutrophil—Lymphocyte Ratio (NLR), (8) Aspartate Aminotransferase (AST)/Serum Glutamic—Oxaloacetic Transaminase (SGOT), 9) Alanine Aminotransferase (ALT)/Serum Glutamic Pyruvic Transaminase (SGPT), (10) bilirubin tests (total, direct, indirect), and (11) sonography cholecystitis findings; intra-operative findings: (1) gallbladder stone count (none, single, multiple) and (2) adhesion; and post-operative evaluations: (1) post-surgery length of stay and (2) microscopic findings (acute or chronic inflammation). All MLC patients filled evaluation questionnaire based on Likert Scale about MLC satisfaction which consisted of incision aspect (size and cosmetic outcome) and post-discharge evaluation (recovery and pain).

This study has been approved by the Institutional Review Board of the Faculty of Medicine, Public Health and Nursing, Universitas Gadjah Mada and Dr. Sardjito Central Hospital, Yogyakarta, Indonesia (KE/FK/0796/EC/2018). This study had been carried out in accordance with all relevant guidelines and regulations. Written informed consent was obtained from all patients for participating this study. The participating patients had also given written consent for the photographs to be used and published in this study.

The inclusion criteria of this study were: (1) the patient only underwent cholecystectomy at that moment, (2) there were no other diseases affected the cholecystitis and surgery procedure. Whereas the exclusion criteria of this study were: 1) the patient didn’t consent for the study, (2) the analyzed data couldn’t be obtained.

The analyses were made with SPSS Statistics 23 (IBM Corp., Armonk, NY). Numerical variables were tested for normality with Kruskal Wallis test. Normally distributed numerical data were compared using independent sample T-test, while other numerical data were compared using Mann–Whitney U test. Categorical data were analyzed using Chi-square test.

## Surgical procedures

**Fig. 3:**
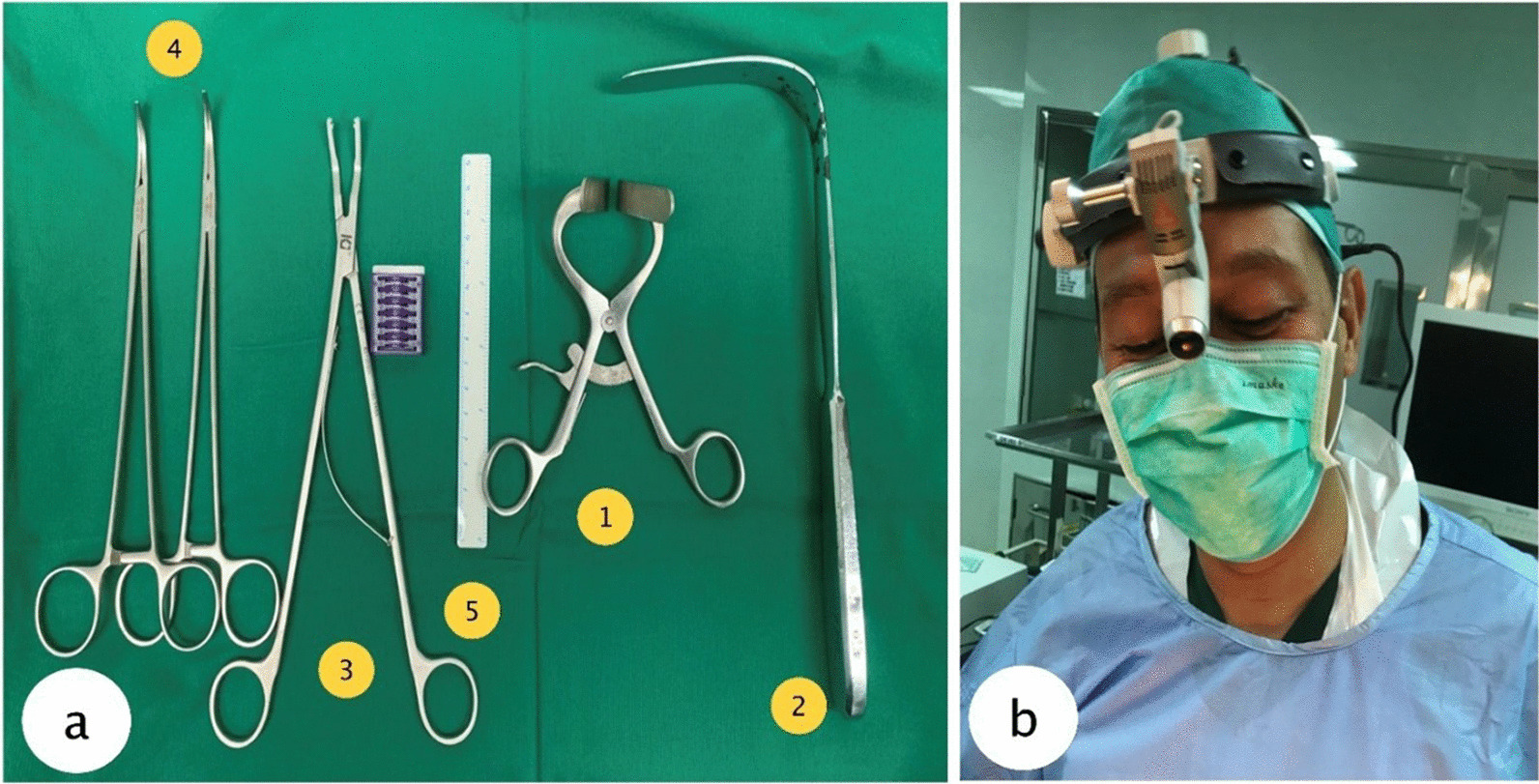
Instruments used in mini-laparotomy cholecystectomy. **a1** Modified Molt dental mouth gag with the prong tip length 5 cm. **a2** Modified Langenbeck retractor with shaft length of 26 cm blade length of 10 cm. **a3** Hem-O-Lok clips and applicator for open surgery. **(a4)** right angle forceps. **a5** ruler for size measurement. **b** Operating surgeon wearing head lamp

1. A right subcostal (Kocher) incision was performed. In the mini laparotomy technique, smaller incision around 4-5 cm was created.
2. Incision was made on the anterior rectus sheath along the length of the incision. Rectus and lateral muscles (external oblique, internal oblique, and transversus abdominis) were divided with the electrocautery.
3. Incisions were made on the posterior rectus sheath and peritoneum to enter the abdomen.
4. The gap was widened and maintained with a specially designed retractor by using a Molt dental mouth gag (Fig. 3.a1) which had been modified.
5. Gallbladder and the surrounding organs were identified.
6. With sterile cotton gauze and specially modified Langenbeck retractor (Fig. 3.a2), the liver and duodenum were displaced inferiorly to expose more of the porta hepatis and the gallbladder.
7. Fundus of the gallbladder was grasped and elevated superiorly while the neck of the gallbladder was mobilized away from the liver laterally to expose the triangle of Calot. Cystic artery and cystic duct were dissected with the help of specially modified right angle artery forceps (Fig. 3.a4).
8. The cystic artery and cystic duct were then clipped with “hem-o-lok” clips using the “hem-o-lok” applicator (Fig. 3.a3) for open surgery.
9. Gallbladder was dissected away from the liver bed. The gallbladder then evacuated out from peritoneal cavity.
10. Bleeding was controlled, if present, and the wound was closed by suturing each incised layer.

## Results

Forty-four patients were included in this study, consisted of 14 male (31.8%) and 30 female (68.2%) with the mean age of 49.57 years old (Tables 1, 2). Conversion from MLC to OC was seen in 13 (29.5%) patients. There were no complications observed during and after the surgery. There were no mortalities occurred in this study.

**Table 1:** Subjects’ characteristics in categorical variables

Variables	No Conversion (n = 31) [70.5%]	Conversion (n = 13) [29.5%]	All Subjects (n = 44) [100%]
Sex			
Male	8 (18.2%)	6 (13.6%)	14 (31.8%)
Female	23 (52.3%)	7 (15.9%)	30 (68.2%)
Cholecystitis Clinical Manifestation			
None	13 (29.5%)	3 (6.8%)	16 (36.4%)
Present	18 (40.9%)	10 (22.7%)	28 (63.6%)
USG Cholecystitis Findings			
None	23 (52.3%)	0	23 (52.3%)
Present	8 (18.2%)	13 (29.5%)	21 (47.7%)
Cholelithiasis			
None	0	0	0
Single stone	11 (25.0%)	5 (11.4%)	16 (36.4%)
Multiple stones	20 (45.5%)	8 (18.2%)	28 (63.6%)
Adhesion			
None	24 (54.5%)	1 (2.3%)	25 (56.8%)
< 50% adhesion	7 (15.9%)	4 (9.1%)	11 (25.0%)
50–100% adhesion	0	5 (11.4%)	5 (11.4%)
Completely buried gallbladder	0	3 (6.8%)	3 (6.8%)
Microscopic findings			
Normal	0	0	0
Acute inflammation	30 (68.2%)	5 (11.4%)	35 (79.5%)
Chronic inflammation	1 (2.3%)	8 (18.2%)	9 (20.5%)

**Table 2:** Subjects’ characteristics in numeric variables and comparison results

Variables	All Subjects (n = 44)		No Conversion (n = 31)		Conversion (n = 13)		P—Value
	Mean	Std. Dev	Mean	Std. Dev	Mean	Std. Dev	
Independent Sample T-Test							
Age (years)	49.57 (26–76)	14.09	48.16 (26–76)	15.42	52.92 (33–67)	9.96	0.312
BMI	25.31 (15.03–40.40)	5.34	24.16 (15.03–40.40)	5.16	28.05 (21.26–38.44)	4.90	0.026**
Hemoglobin Level (g/dL)	12.93 (9.9–16.7)	1.35	12.90 (9.9–16.7)	1.48	12.99 (11.5–14.6)	1.04	0.852
Leucocytes Count (× 10^9^/L)	8.66 (3.70–15.60)	2.84	7.78 (3.70–13.20)	2.16	10.75 (6.40–15.60)	3.23	0.001**
Indirect Bilirubin (mg/dL)	0.46 (0.08–1.10)	0.21	0.41 (0.1–0.7)	0.17	0.59 (0.08–1.10)	0.25	0.008**
Mann Whitney U Test							
Neutrophil—Lymphocyte Ratio*	3.34 (0.95–21.44)	3.79	2.29 (0.95–8.06)	1.25	5.85 (1.71–21.44)	6.17	0.052
AST / SGOT* (U/L)	20.57 (6–46)	7.99	20.84 (6–46)	8.50	19.92 (12–35)	6.89	0.651
ALT / SGPT* (U/L)	27.49 (11–96)	20.09	28.31 (11.0–96.0)	20.77	25.54 (12–76)	19.03	0.303
Total Bilirubin* (mg/dL)	0.81 (0.3–2.2)	0.34	0.72 (0.3–1.2)	0.26	1.02 (0.6–2.2)	0.42	0.015**
Direct Bilirubin* (mg/dL)	0.36 (0.1–1.1)	0.19	0.32 (0.1–0.7)	0.18	0.43 (0.20–1.10)	0.23	0.049**
Adhesion							0.000**
Gall Bladder Stone Count							0.853
Post-Surgery Hospitalization* (days)	2.43 (2–5)	0.73	2.10 (2–3)	0.30	3.23 (2–5)	0.83	0.000**
Surgery Duration (minutes)*	42.61 (20–75)		38.87 (20–75)	10.22	51.54 (30–70)	10.87	0.001**
Chi Square Test							
Gender							0.166
Cholecystitis Clinical Manifestation							0.201
USG Cholecystitis Findings							0.000**
Microscopic Findings							0.000**

*Data not normally distributed, Kruskal Wallis Test

**P-value < 0.05

In the comparison of non-conversion and conversion group, we found no difference in age and gender. However, the mean BMI was greater in the conversion group with p-value of 0.026. The were no difference in conversion risk in term of cholecystitis clinical manifestation, but there was greater risk of conversion if cholecystitis was found in sonography examination (p-value 0.000). Pre-operative laboratory examination showed no difference in conversion risk in hemoglobin level, but mean leucocytes count was higher in conversion group (p-value 0.001), as well as all bilirubin measurement. Despite the difference in leucocyte count, there were no difference in neutrophil—lymphocytes ratio between the two groups, and there also no difference in liver function examination.

In intra-operative variables, we found same risk of conversion if single or multiple gallstones were found in the surgeries, but higher conversion risk along with higher degree of adhesion (p-value 0.000). In post-operatives’ variables analyses, we found longer mean post-operative care for conversion group (p-value 0.000) and pathology findings showed chronic cholecystitis possessed higher risk of conversion in mini laparotomy cholecystectomy (p-value 0.000).

Using Likert Scale Questionnaire, MLC subjects showed great satisfaction (above 4.0 out of 5.0 mean score) towards the result of the procedure in incision and post-discharge evaluation of MLC procedures (Table 3).

**Table 3:** Evaluation of patient’s satisfaction towards the mini-laparotomy cholecystectomy using Likert Scale Questionnaire. 5-point Likert scale: (1) Strongly dissatisfied; (2) Dissatisfied; (3) Neutral; (4) Satisfied; (5) Very satisfied

Patient’s satisfaction towards mini-laparotomy cholecystectomy		Likert Score	
		Mean	Std. Dev
Incision	Size of post-operative wound	4.39	0.72
	Cosmetic outcome of post-operative wound	4.39	0.89
Post-discharge	Post-discharge time to recovery	4.48	0.88
	Post-discharge pain related to surgery	4.57	0.76

## Discussion

In this study, 31 (70.5%) subjects underwent MLC, while the other 13 (29.5%) subjects underwent conversion surgery. Most conversion was decided during surgery due to adhesion of gallbladder more than 50% and difficult access due to thick fat layer on higher BMI patients. Based on post-surgery microscopic finding, most patients who underwent conversion surgery had chronic inflammation in the gallbladder. Chronic inflammation resulted in changes of the organ wall that may also affect the surrounding organs. This finding supports the incidence of gallbladder adhesion. In the analysis, we found that the degree of adhesion is statistically significant to conversion surgery. Thus, severity of adhesion and chronic inflammation have been proven to be the predictors of difficult cholecystitis surgery which may require a conversion surgery. Gallbladder adhesion is also related to many intra-surgery complications such as organ injury and perforation during the dissection due to inability to define clear anatomy [5, 6]. Gallbladder adhesion and chronic inflammation have been observed to be a predictor of conversion surgery both in external study and in our study.

There are other factors that are statistically significant in the relation with conversion surgery. Higher BMI, higher bilirubin level, and higher leucocytes count were also observed to be predictors of conversion surgery and has been shown to be significant. Elevated leukocyte count is already reported in many studies to be a predictor of conversion surgery. The high leukocyte number often indicate the severity of inflammation in the gallbladder which is also correlated with the increasing possibility of other comorbidities [6]. High bilirubin level has also been reported in few studies as a risk factor of conversion surgery. High bilirubin level shows a strong indication of biliary duct obstruction which often found to be choledocholithiasis. An abnormally high bilirubin level with high SGOT/SGPT further indicate an extensive inflammation process in the biliary system such as cholangitis and obstruction most likely due to inflamed organ surrounding the ducts [4, 7].

In many reports, MLC is less difficult to learn when compared to LC and reported to have a shorter operating time by approximately 14–25 min [8, 9]. Few studies reported that patients in LC group experienced less pain as compared to their counterparts in the MLC group (median of 2 days and 3 days for LC group and MLC group consecutively), however the difference was not found to be statistically significant [10, 11]. In our study, it was observed that patients that underwent MLC had shorter length of hospital stay after surgery with an average LOS of 2 days compared to average LOS of 3 days in the conversion surgery group. The analysis has proven that shorter LOS in MLC group to be statistically significant. Furthermore, all patients that underwent MLC showed a great satisfaction towards result of MLC consisting of cosmetic aspects and recovery period after surgery. Currently, MLC is the preferred method to be performed by new surgeons and in cases of difficult cholecystectomy [8, 9].

Considering the financial situation in developing countries, cost analysis is always an important factor. MLC seems to be the preferred operative technique over the laparoscopic technique both from a hospital and societal cost perspective. In South Africa, Calvert et al. reported that MLC costs 26% less than LC with cost of equipment and operations themselves accounting for most of the difference [5]. In the two hospitals where we did this study, we found that the average operational cost of LC is IDR 17.8 million (USD 1250) while the average cost of MLC is IDR 12.8 million (USD 900). This shows that LC is currently 38% costlier than MLC. When accumulated, this 38% cost increase will become a financial toll to the government. Therefore, MLC is much more affordable than LC, thus allowing more provision of affordable health care services.

Surgical instruments used in MLC are mostly affordable. However, these instruments came in inadequate length resulting in difficulty in some cases, especially in obese patients, when the blade or tip is insufficient to properly retract the skin and subcutaneous tissue [12, 13]. Therefore, we modified few tools to suit the MLC procedures better during the period of this study. Modifications of size and thickness of the tools are needed to ease the visualization and prevent instrumental damage in MLC procedure, especially in obese patients. For example, a Molt dental mouth gag that can be commonly found (Figs. 3.a1, 4) was modified so that the prong tip is longer to reach all abdominal layer, therefore allowing the mouth gag to become a surgical retractor for the small incision. Another tool that has been modified is the Langenbeck retractor (Fig. 3.a2). The retractor is modified to have a longer shaft and blade. The modified retractor is able to hold abdominal organs through the small incision while the conventional Langenbeck retractor is usually used to hold only the soft tissue and wound edge. The modification of these tools was not difficult, therefore allowing MLC very feasible in any settings. Other tools, such as ruler (Fig. 3.a5) and headlamp (Fig. 3b) are using regular type which widely available. These tools can be purchased each for USD 10–15 and the modification cost below USD 10 for each tool.

**Fig. 4:**
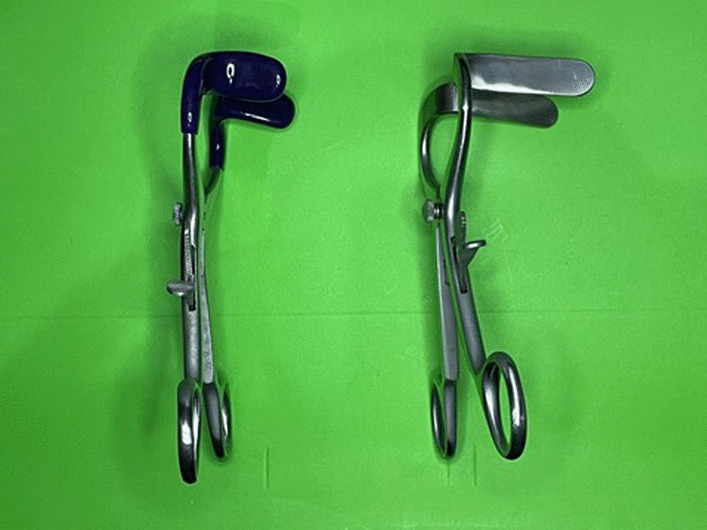
(left) Unmodified dental mouth gag. (right) Modified dental mouth gag used for mini laparotomy cholecystectomy

## Conclusion

MLC is a safe and effective alternative surgery procedure with shorter post-operative hospitalization, better cosmetic result, and shorter recovery period. The conversion risk of increase in leucocyte count, bilirubin level, USG cholecystitis finding, adhesion, and chronic inflammation pathologic finding should be taken as precaution for the surgeons.

## Data Availability

All data generated or analyzed during this study are included in this published article.
